# Basal fatty acid oxidation increases after recurrent low glucose in human primary astrocytes

**DOI:** 10.1007/s00125-018-4744-6

**Published:** 2018-10-06

**Authors:** Paul G. Weightman Potter, Julia M. Vlachaki Walker, Josephine L. Robb, John K. Chilton, Ritchie Williamson, Andrew D Randall, Kate L. J. Ellacott, Craig Beall

**Affiliations:** 1 Institute of Biomedical and Clinical Sciences, University of Exeter Medical School, RILD Building, Barrack Road, Exeter, EX2 5DW UK; 2 School of Pharmacy and Medical Sciences, University of Bradford, Bradford, UK; 3 Hatherly Laboratories, Prince of Wales Road, University of Exeter, Exeter, UK

**Keywords:** Adenosine triphosphate, AMP-activated protein kinase, Astrocyte, Diabetes, Fatty acid oxidation, Glia, Hypoglycaemia, Lactate, Low glucose, Mitochondrial metabolism

## Abstract

**Aims/hypothesis:**

Hypoglycaemia is a major barrier to good glucose control in type 1 diabetes. Frequent hypoglycaemic episodes impair awareness of subsequent hypoglycaemic bouts. Neural changes underpinning awareness of hypoglycaemia are poorly defined and molecular mechanisms by which glial cells contribute to hypoglycaemia sensing and glucose counterregulation require further investigation. The aim of the current study was to examine whether, and by what mechanism, human primary astrocyte (HPA) function was altered by acute and recurrent low glucose (RLG).

**Methods:**

To test whether glia, specifically astrocytes, could detect changes in glucose, we utilised HPA and U373 astrocytoma cells and exposed them to RLG in vitro. This allowed measurement, with high specificity and sensitivity, of RLG-associated changes in cellular metabolism. We examined changes in protein phosphorylation/expression using western blotting. Metabolic function was assessed using a Seahorse extracellular flux analyser. Immunofluorescent imaging was used to examine cell morphology and enzymatic assays were used to measure lactate release, glycogen content, intracellular ATP and nucleotide ratios.

**Results:**

AMP-activated protein kinase (AMPK) was activated over a pathophysiologically relevant glucose concentration range. RLG produced an increased dependency on fatty acid oxidation for basal mitochondrial metabolism and exhibited hallmarks of mitochondrial stress, including increased proton leak and reduced coupling efficiency. Relative to glucose availability, lactate release increased during low glucose but this was not modified by RLG. Basal glucose uptake was not modified by RLG and glycogen levels were similar in control and RLG-treated cells. Mitochondrial adaptations to RLG were partially recovered by maintaining euglycaemic levels of glucose following RLG exposure.

**Conclusions/interpretation:**

Taken together, these data indicate that HPA mitochondria are altered following RLG, with a metabolic switch towards increased fatty acid oxidation, suggesting glial adaptations to RLG involve altered mitochondrial metabolism that could contribute to defective glucose counterregulation to hypoglycaemia in diabetes.

**Electronic supplementary material:**

The online version of this article (10.1007/s00125-018-4744-6) contains peer-reviewed but unedited supplementary material, which is available to authorised users.

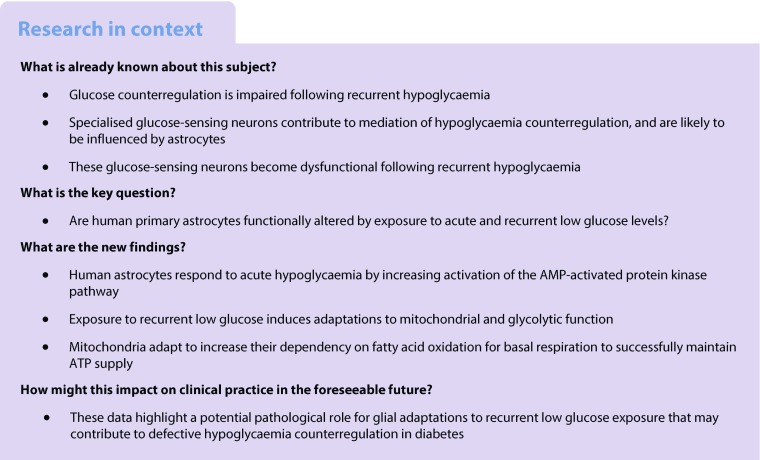



## Introduction

Hypoglycaemia remains a major concern for people with type 1 diabetes and advanced insulin-treated type 2 diabetes. For individuals with diabetes, hypoglycaemia is a major barrier to enjoying the benefits of exercise [1, 2] and is increasingly thought to increase the risk of cardiovascular events in both type 1 and type 2 diabetes [3, 4]. Individuals who experience frequent hypoglycaemia or have long-duration diabetes produce diminished counterregulatory responses (CRRs) to hypoglycaemia [5, 6], often accompanied by reduced symptom awareness, termed ‘impaired hypoglycaemia awareness’.

Defective glucose CRRs to hypoglycaemia are, at least in part, mediated by changes in the function of critically important glucose-sensing neurons in the ventromedial nucleus of the hypothalamus (VMH), although glucose-sensing neurons in the hindbrain have also been implicated [7]. In addition to neurons, the brain contains at least as many cells that lack electrically excitable membranes. Of these, the largest group is the glial cells, the largest subpopulation of which are astrocytes. As well as exhibiting a range of cell-to-cell signalling pathways, astrocytes are increasingly recognised as important players in central nervous system-based diseases, such as obesity [8], Alzheimer’s disease [9], Parkinson’s disease [10] and motor neuron disease [11].

Astrocytes play an important role in regulating glucose uptake into cerebral microvessels [12]. In addition to this role in glucose delivery, more recent studies have demonstrated that stimuli such as 2-deoxyglucose, that block intracellular glucose metabolism, increase intracellular Ca^2+^ in hindbrain astrocytes [13], a well-accepted marker of increased astrocyte activity, suggesting a more direct role in sensing changes in glucose levels. In the hypothalamus, astrocyte activation by fasting is indicated by increased glial fibrillary acidic protein (GFAP) expression and process complexity [14, 15], both markers of gliosis.

Astrocytes contain small amounts of glycogen that can be broken down to glucose and metabolised to lactate, which is released to feed active neurons. For example, astrocytic glycogen stores can maintain axonal activity during energetic stress in white matter [16, 17] and also contribute to memory formation [18, 19]. Brain glycogen concentrations are increased on recovery from exercise [20], suggesting that astrocytic glycogen stores are both physiologically and pathophysiologically regulated. Astrocytes are in intimate apposition to the cerebral vasculature, meaning that a change in glycaemia may be detected by astrocytes before neurons. Moreover, astrocytes are generally accepted as metabolically flexible neural cells that can rapidly upregulate glycolysis to support neuronal function, partly through the higher expression of 6-phosphofructo-2-kinase type 3 (PFKFB3), which can stimulate glycolysis [21]. Despite this, little is known about the intrinsic response of astrocytes to acute and recurrent low glucose (RLG) that occurs in type 1 diabetes.

Given that astrocytes are metabolically flexible cells, we hypothesised that human astrocytes would: (1) sense low glucose by increasing phosphorylation of cellular energy sensor AMP-activated protein kinase (AMPK); and (2) exhibit altered glycolytic and mitochondrial metabolism following RLG. To test this, we utilised both human primary astrocytes (HPA) and U373 astrocytoma cells and measured directly components of glycolytic and mitochondrial metabolism, lactate release, glycogen levels, intracellular nucleotide levels and expression of key metabolic enzymes.

## Methods

### Astrocyte isolation and cell culture

HPA were isolated from a 17 year old male donor from subventricular deep white matter tissue immediately post-mortem following consent from next of-kin and with ethical approval from the North and East Devon Research Ethics Committee. Astrocytes were isolated from tissue blocks as previously described [22], subcultured and frozen stocks prepared. HPA cells were maintained in humidified incubators with 95% O_2_/5% CO_2_ in HPA stock media. U373 cells were obtained from American Type Culture Collection (ATCC, Manassas, VA, USA). See electronic supplementary material (ESM) Methods for further details of stock media components.

For experiments, the glucose concentration was reduced to 2.5 mmol/l glucose for 2 h, supplemented with 1% serum and 22.5 mmol/l mannitol for HPA cells. Glucose concentrations were then dropped further to 0.1 mmol/l glucose as a hypoglycaemic-like stimulus or plated at 2.5 mmol/l glucose, supplemented with 1% (vol./vol.) serum and 24.9 mmol/l or 22.5 mmol/l mannitol, for HPA and U373 cells, respectively. For RLG studies over 4 days, control cells were exposed to zero bouts (2.5 mmol/l glucose instead) and RLG cells exposed to 4 bouts of 3 h of 0.1 mmol/l glucose and recovered in media containing high (HPA, 5.5 mmol/l; U373, 7.5 mmol/l) glucose and FBS overnight (see ESM Fig. 1 for culture model and ESM Methods for further details). The reasons for this were twofold: (1) higher glucose levels overnight (approximately 19 h) prevented glucose depletion from the media, which would occur if the cells were maintained at 2.5 mmol/l; and (2) this pattern more accurately represented a model of glucose variation seen in type 1 diabetes.

All cell lines used were verified as mycoplasma-free using a commercial test kit (MycoAlert, Lonza, Slough, UK). Cells were randomly assigned to experimental groups by an unblinded investigator (unless otherwise stated).

### Immunoblotting

Cellular protein was isolated as previously described [23], with minor modifications. Briefly, for acute studies 4.5 × 10^5^ cells or, for RLG studies, 2.5 × 10^5^ cells were seeded on to 60 mm petri dishes and harvested in 65 μl lysis buffer. Protein concentrations were assessed by Bradford assay. A 10 μg sample of protein was loaded per lane on 7–10% (wt/vol.) polyacrylamide gels. Proteins were transferred to nitrocellulose membranes, blocked with BSA (5% wt/vol.) or powdered milk (5% wt/vol.) and probed with antibodies against target proteins. See ESM Methods for antibody details.

### Analysis of cellular metabolism

HPA and U373 cells were seeded at 2 × 10^4^ cells per well in Seahorse XF^e^96 assay plates (no. 102416-100, Agilent, Craven Arms, UK) on the day before the study. Medium was exchanged for low-buffered media, the pH adjusted to 7.40 at 37.0°C and cells placed in an atmospheric CO_2_ incubator, to remove CO_2_ buffering capacity, 1 h prior to starting the assay. Mitochondrial stress tests (no. 103015-100, Agilent) and mitochondrial fuel flexibility tests (no. 103270-100, Agilent) were performed as per manufacturer’s instructions (see ESM Methods for further details). Samples with negative values for OCR (probably due to the presence of an air bubble) were excluded from analysis.

### Measurement of intracellular nucleotides

Total ATP levels were measured using ATPlite (no. 6016941, Perkin Elmer, Seer Green, UK) with minor modifications as previously described [23]. ATP/ADP ratios were assayed by luminescence assay (no. MAK135, Sigma-Aldrich, Gillingham, UK). Briefly, 1 × 10^3^ cells were seeded on to black-walled 96 well plates and exposed to 2.5 mmol/l or 0.1 mmol/l glucose levels for 15–180 min and nucleotide ratios assayed as per manufacturer’s instructions.

### Measurement of extracellular lactate, glucose uptake and glycogen levels

Extracellular lactate was measured by assessing NADH production from NAD^+^ in the presence of lactate dehydrogenase as previously described [24]. Briefly, 100 μl of extracellular supernatant fraction was examined against a standard curve of lactate from 0 nmol/l to 50 nmol/l. Glucose uptake was measured using fluorescently labelled glucose analogue 6-(*N*-(7-nitrobenz-2-oxa-1,3-diazol-4-yl)amino)-6-deoxyglucose (6-NBDG; CAY13961-5/6-NBDG, Cayman Chemical, Tallinn, Estonia). 6-NBDG (600 μmol/l) was added for 15 min in 2.5 mmol/l glucose in control conditions and in cells exposed to RLG. Glycogen levels were measured using a fluorometric kit as per manufacturer’s instructions (no. MET-5023, Cambridge Bioscience, Cambridge, UK).

### Fluorescent imaging

Mitochondrial morphology was examined by staining HPA cells with MitoTracker Red CMXRos (50 nmol/l; M7512, no. 1785958, Thermo Fisher Scientific, Loughborough, UK) before fixing and imaging using confocal microscopy (Leica, London, UK DMi8; ×63/oil immersion lens) by an investigator blind to sample identity. See ESM Methods for further details. To examine intermediate filament protein expression/localisation, HPA cells were fixed and stained with an anti-vimentin antibody (V6630, no. 102M4831, mouse, Sigma-Aldrich; 1:500 in lysine buffer) or an anti-GFAP antibody (z0334 no. 20035994, rabbit, Agilent; 1:100 in lysine buffer). All antibodies were validated by the manufacturer. See ESM Methods for further details.

### Statistical analysis

For immunoblotting, densitometric values were normalised to unity to examine relative fold change in expression. A one-sample *t* test was used to determine significant changes in phosphorylation or expression, relative to control. For comparisons of two groups a two-tailed unpaired *t* test was used and for multiple group comparisons, a one-way ANOVA with post hoc Bonferroni were used. To compare the mean differences between groups split by two independent variables, a two-way ANOVA with Bonferroni multiple comparisons tests was used. Statistical tests were performed using GraphPad Prism software (Prism 5; GraphPad Software, La Jolla, CA, USA). Results are expressed as mean ± standard error, unless otherwise stated. Values of *p* < 0.05 were considered statistically significant.

## Results

### AMPK is activated by acute low glucose in human astrocytes

**Fig. 1:**
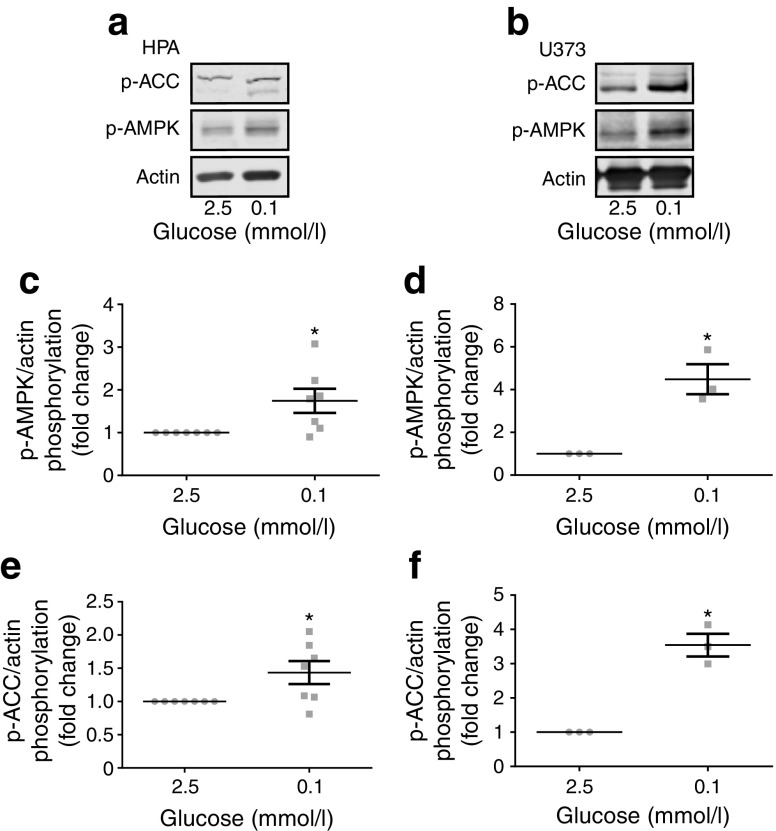
Acute low glucose increased AMPK pathway activation. Representative immunoblots from HPA (**a**; *n* = 8) and U373 astrocytoma cells (**b**; *n* = 3) exposed to 2.5 or 0.1 mmol/l glucose media for 30 min. (**c**, **d**) Densitometric analysis of immunostaining for pT172 AMPK normalised to actin as a loading control in HPA (**c**) and U373 astrocytoma cells (**d**). (**e**, **f**) Densitometric analysis of immunostaining for pS79 ACC normalised to actin as a loading control in HPA (**e**) and U373 astrocytoma cells (**f**). Error bars represent SEM. *p < 0.05, one-sample *t* test vs control (2.5 mmol/l glucose)

AMPK activation in neurons is required for sensing hypoglycaemia [25], though it is not known whether astrocytic AMPK reacts to the same energy stress. To address this, we exposed human astrocytes to normal (2.5 mmol/l) and low (0.1 mmol/l) glucose levels for brain cells. In cells exposed to low glucose for 30 min, we noted, in both the HPA (Fig. 1a, c) and U373 cells (Fig. 1b, d), an increased phosphorylation of AMPK at threonine 172, a site which must be phosphorylated for full kinase activation. This was accompanied by increased phosphorylation of acetyl-CoA carboxylase (ACC) at serine 79 (Fig. 1a, b, e, f). These novel observations in glia are consistent with the ubiquitous role of AMPK in detecting low glucose levels in other cells types. In contrast, although the astrogliosis marker GFAP was expressed in HPA cells, its expression appeared unaltered in cells exposed to acute low glucose (ESM Fig. 2).

### RLG exposure increases mitochondrial metabolism in human astrocytes

To determine whether baseline metabolism was altered following RLG, we performed mitochondrial stress tests on HPA and U373 cells (see ESM Fig. 1 for RLG model). In order to observe any cellular adaptations, this was performed in 2.5 mmol/l glucose, representing euglycaemic, glucose levels following RLG.

**Fig. 2:**
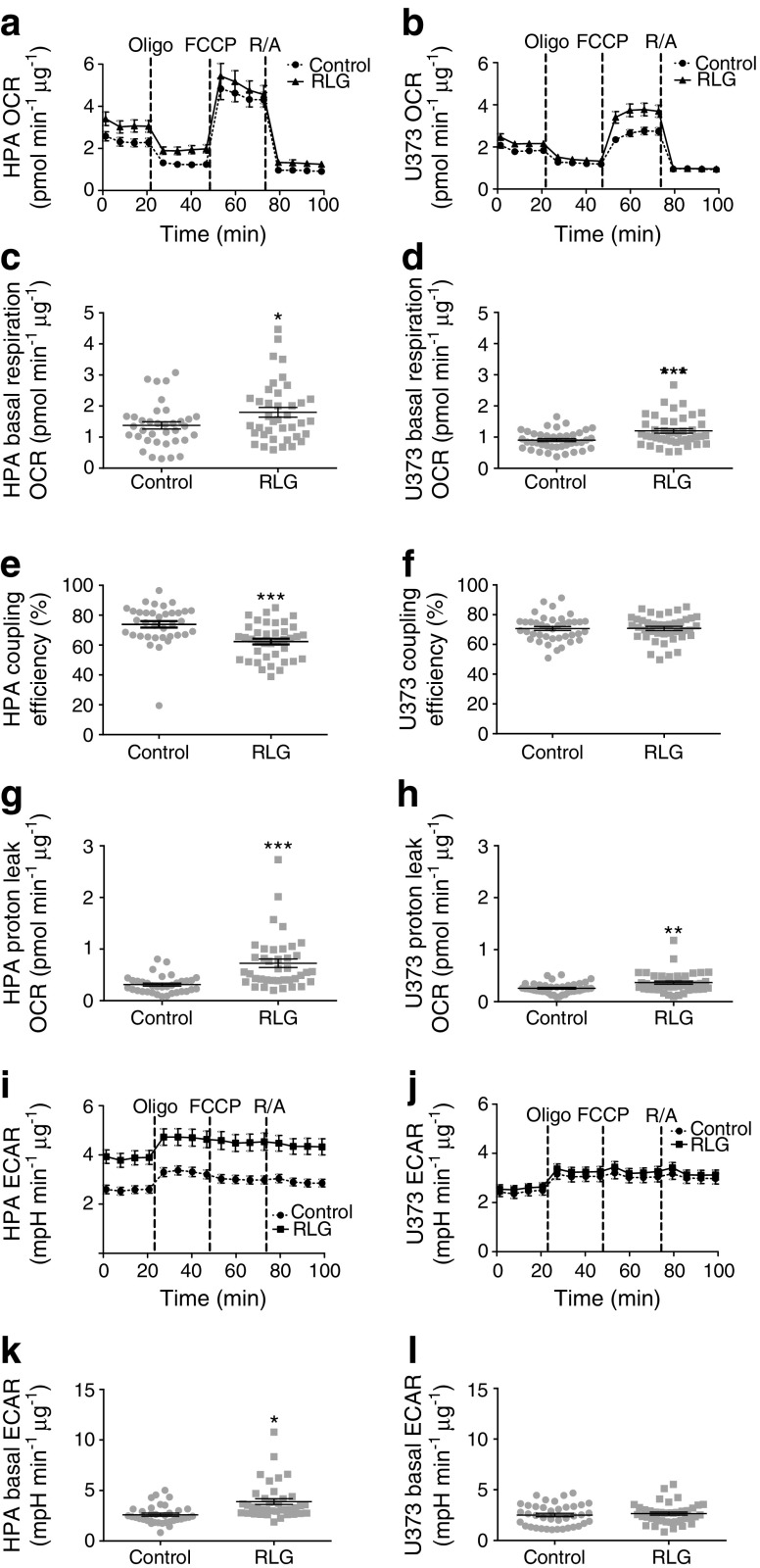
RLG increased baseline mitochondrial metabolism and reduced mitochondrial coupling efficiency in HPA (**a**,**c**,**e**,**g**,**i**,**k**) and U373 astrocytoma cells (**b**,**d**,**f**,**h**,**j**,**l**). (**a**, **b**) OCR of HPA (**a**; control, *n* = 36; RLG, *n* = 39, across three separate assays) and U373 cells (**b**; control, *n* = 40; RLG, *n* = 43, across three separate assays) following RLG. Cells were exposed to oligomycin (10 μmol/l), FCCP (5 μmol/l) and a combination of rotenone and antimycin A (5 μmol/l). (**c**, **d**) Mean basal OCR in control and RLG-treated astrocytes. (**e**, **f**) Coupling efficiency calculated from the ratio of oligomycin-sensitive OCR and basal OCR expressed as a percentage. (**g**, **h**) Proton leak, calculated from the oligomycin-insensitive (i.e. not ATP synthase linked) OCR minus non-mitochondrial respiration. (**i**, **j**) ECAR analysis measured during mitochondrial stress tests. (**k**, **l**) Mean baseline ECAR following RLG. Error bars represent SEM. *p < 0.05, **p < 0.01 and ***p < 0.001, unpaired two-tailed student’s *t* test vs control. mpH, milli-pH; Oligo, oligomycin; R/A, rotenone and antimycin A

First, we observed significantly higher baseline oxygen consumption rate (OCR) in both HPA (Fig. 2a, c) and U373 cells (Fig. 2b, d) exposed to RLG (3 vs 0 prior bouts of low glucose), indicating persistent adaptations in cellular metabolism. Oligomycin was then added to block ATP synthase, carbonyl cyanide 4-(trifluoromethoxy)phenylhydrazone (FCCP) to stimulate maximal respiration, and rotenone and antimycin A were added to inhibit complex I and III, respectively. This allowed calculation of coupling efficiency, which was significantly decreased in HPA cells only (Fig. 2e, f). However, both cell types displayed increased proton leak (Fig. 2g, h), indicating mitochondrial dysfunction. Despite the evidence of mitochondrial dysfunction, we saw no changes in the morphology of the filamentous mitochondrial networks between the treatment groups, as examined using MitoTrackerRed (ESM Fig. 3a, b).

In addition to changes in OCR, we also noted significantly elevated basal extracellular acidification rate (ECAR), in HPA cells exposed to RLG (Fig. 2i, k), indicating enhanced glycolysis following RLG. This did not occur within U373 cells following RLG (Fig. 2j, l).

### Astrocytes exposed to RLG display enhanced glycolysis on recovery of glucose level

**Fig. 3:**
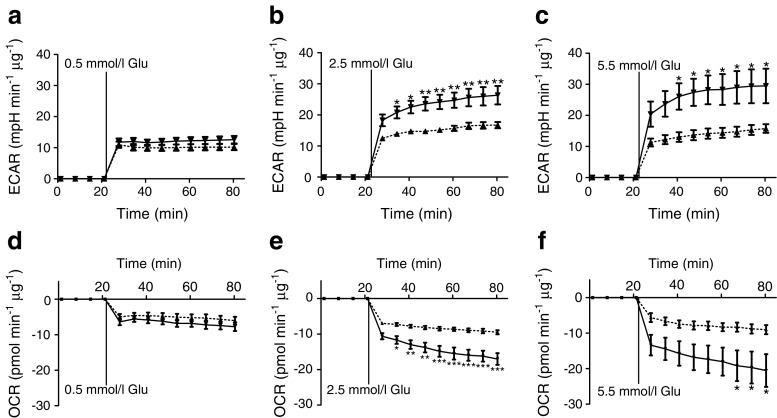
RLG increased glycolytic re-activation after acute low glucose exposure. (**a**–**c**) ECAR increased in HPA cells on re-introduction of 0.5 mmol/l glucose (**a**), 2.5 mmol/l glucose (**b**) and 5.5 mmol/l glucose (**c**). (**d**–**f**) OCR decreased on addition of 0.5 mmol/l glucose (**d**), 2.5 mmol/l glucose (**e**) and 5.5 mmol/l glucose (**f**). Dashed line, control; solid line, RLG. Control, *n* = 8–10; RLG, *n* = 9–10. Error bars represent SEM. *p < 0.05; **p < 0.01 and ***p < 0.001, unpaired two-tailed student’s *t* test vs control. Glu, glucose; mpH, milli-pH

Given the increased basal ECAR in HPA cells exposed to RLG, we next examined glycolytic re-activation after an acute glucose withdrawal (~80 min) in HPA cells following prior exposure to RLG. Under control conditions, we noted that the ECAR (glycolysis) response to glucose was concentration dependent (Fig. 3a–c), although ECAR was not significantly higher at 5.5 mmol/l glucose than 2.5 mmol/l glucose, indicating that the metabolic response to glucose is saturated at physiological glucose levels. Interestingly, the ECAR response to glucose in cells exposed to RLG (three bouts of low glucose) was significantly greater than control (no bouts of low glucose), indicating an enhanced glycolytic capacity (Fig. 3a–c). This only occurred at euglycaemic and hyperglycaemic levels. Consistent with enhanced glycolytic activity, we also noted a significant reduction in OCR following glucose injection, indicative of a shift away from mitochondrial metabolism towards glycolysis (Fig. 3d–f). Correspondingly, the reduction in OCR following glucose recovery was augmented in HPA cells exposed to RLG. Taken together, these data suggest an enhanced Warburg-like effect in HPA cells following recovery of glucose levels. In U373 cells, RLG did not alter ECAR between groups (control vs RLG) on glucose recovery. However, the re-activation of ECAR on delivery of glucose was not concentration dependent and in U373 cells the relative increase in ECAR was twice as large as that in HPA cells (ESM Fig. 4). These studies highlight clear differences between primary cells, obtained from source, with those of transformed cells which should be taken into consideration when designing experiments.

### Fatty acid oxidation is increased in human astrocytes following RLG

**Fig. 4:**
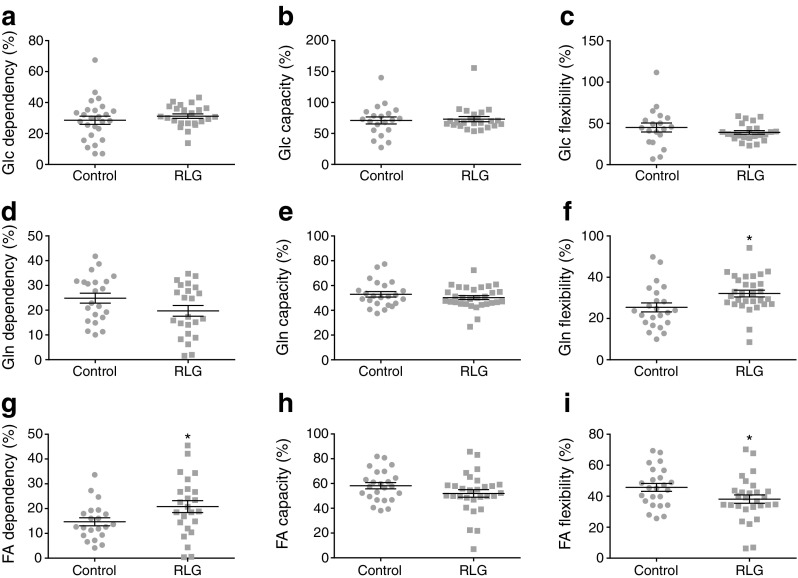
RLG altered HPA mitochondrial fuel flexibility to increase dependency on fatty acids for basal oxidation. (**a**–**c**) Inhibition of MPC using UK5099 (2 μmol/l) to measure dependency (**a**), maximum capacity (**b**) and flexibility (**c**) within the glucose oxidation pathway. (**d**–**f**) Inhibition of glutaminase using BPTES (bis-2-(5-phenylacetamido-1,3,4-thiadiazol-2yl)ethyl sulfide; 3 μmol/l) to measure dependency (**d**), maximum capacity (**e**) and flexibility (**f**) in the glutamine oxidation pathway following RLG. (**g–i**) Inhibition of CPT1 using etomoxir (4 μmol/l) to measure dependency (**g**), maximum capacity (**h**) and flexibility (**i**) in the FAO pathway following RLG. Dependency: contribution of that pathway to baseline OCR. Capacity: maximum ability to oxidise through that pathway when two other oxidative pathways are inhibited. Flexibility: calculated from the difference between dependency and capacity. Control, *n* = 21; RLG, *n* = 24. Error bars represent SEM. *p < 0.05, unpaired two-tailed Student’s *t* test vs control. FA, fatty acid; Glc, glucose; Gln, glutamine

To assess potential changes to mitochondrial fuel usage following RLG, we performed mitochondrial fuel flexibility tests, achieved by sequential selective inhibition of mitochondrial pyruvate carrier (MPC; glucose oxidation pathway), glutaminase (glutamine oxidation pathway) and carnitine palmitoyltransferase 1 (CPT1; fatty acid oxidation pathway). Surprisingly, there was no change in mitochondrial dependency, capacity or flexibility to metabolise pyruvate in HPA cells (Fig. 4a–c). This suggests that in astrocytes, alterations to glucose metabolism are upstream of the transport of pyruvate into the mitochondria. There was a non-significant trend towards reduced glutamine dependency at baseline following RLG (Fig. 4d), with no significant change to the glutamine oxidation capacity (Fig. 4e). Combined, this produced a significant increase in glutamine flexibility (Fig. 4f). Interestingly, RLG significantly increased metabolic dependency on fatty acids but with no change in fatty acid oxidation (FAO) maximum capacity, thereby causing a decrease in flexibility (Fig. 4g–i). These data demonstrate that RLG induces mitochondrial adaptations to fuel sources, consistent with a shift towards increased reliance on FAO and an enhanced flexibility to utilise glutamine.

### Human astrocytes maintain energy production (total ATP and ATP/ADP ratio) during low glucose conditions

**Fig. 5:**
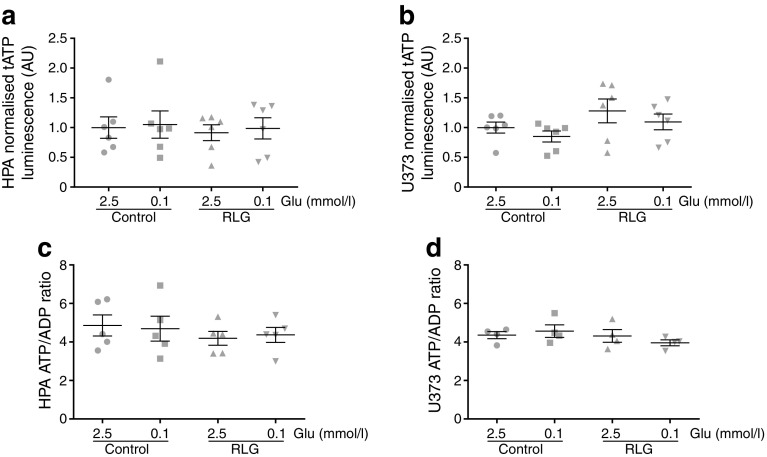
Acute and recurrent low glucose did not modify intracellular ATP or ATP/ADP ratios. (**a**, **b**) Total intracellular ATP levels in HPA (**a**, *n* = 6) and U373 astrocytoma cells (**b**; *n* = 6) exposed to different glucose concentrations for 3 h. (**c**, **d**) Intracellular ATP/ADP ratios in HPA (**c**; *n* = 6) and U373 astrocytoma cells (**d**; *n* = 4) exposed to 2.5 or 0.1 mmol/l glucose for 3 h. One-way ANOVA with post hoc Bonferroni multiple comparisons tests. Glu, glucose; tATP, total ATP

To examine the consequences of acute low glucose and RLG for astrocyte energy content, we measured total ATP and ATP/ADP ratios. These measurements were made both in the presence of acute low glucose and following RLG. Interestingly, total ATP levels did not significantly change in HPA (Fig. 5a) or U373 cells (Fig. 5b) during acute low glucose exposure (0.1 mmol/l) lasting 3 h. Despite the increased basal mitochondrial and glycolytic metabolism described above, total ATP levels following RLG were also comparable with control (Fig. 5a, b). Similarly, the ATP/ADP ratio was not significantly different following 3 h of 0.1 mmol/l glucose, nor was the basal ratio altered following RLG (Fig. 5c, d). These data indicate that the total ATP content and ATP/ADP ratio are well maintained in astrocytes, at least in response to up to 3 h of low glucose, and that changes to glycolytic and mitochondrial metabolism may be compensatory to sustain intracellular nucleotide levels.

### RLG does not alter glucose uptake or glycogen levels in human astrocytes

**Fig. 6:**
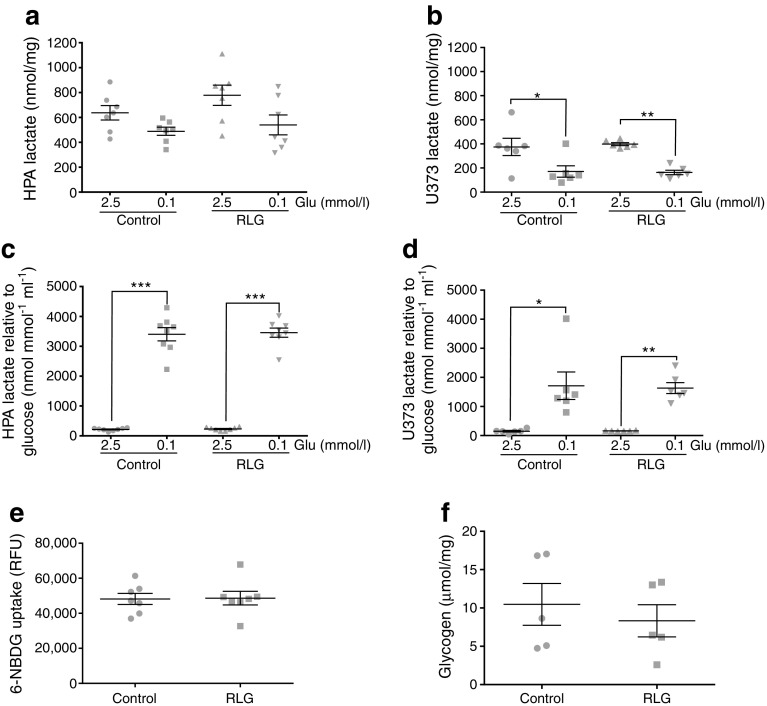
RLG does not alter lactate release, glucose uptake or glycogen levels in HPA. (**a**, **b**) Extracellular lactate levels measured from conditioned media from HPA (**a**; *n* = 8) and U373 (**b**; *n* = 6) following exposure to 2.5 or 0.1 mmol/l glucose-containing media for 3 h. (**c**, **d**) Extracellular lactate levels normalised to glucose availability from conditioned media from HPA (**c**; *n* = 8) and U373 (**d**; *n* = 6) following exposure to 2.5 or 0.1 mmol/l glucose-containing media for 3 h. (**e**) Fluorescent signal from labelled glucose analogue 6-NBDG incubated with HPA cells for 15 min. (**f**) Glycogen content of HPA cells in control and following exposure to RLG (*n* = 5). *p < 0.05, **p < 0.01 and ***p < 0.001, one-way ANOVA with post hoc Bonferroni multiple comparisons tests, and unpaired two-tailed Student’s *t* test. RFU, relative fluorescence units

Western blotting demonstrated that the levels of key glycolytic markers GLUT1, hexokinase (HK)I, HKII, HKIII and phosphofructokinase [platelet] (PFK[P]) were not altered by acute or RLG in human astrocytes (ESM Fig. 5). Lactate release modestly decreased from HPA cells and significantly decreased from U373 cells during 3 h of low glucose exposure (Fig. 6a, b), although the decrease was proportionally less than the 25-fold reduction in glucose availability. Hence, when corrected for glucose availability, there was a relative increase in lactate release (Fig. 6c, d); however, this was not different between control and RLG-treated cells.

We next assessed the degree of glucose uptake by human astrocytes using the fluorescently labelled glucose analogue, 6-NBDG. HPA cells exposed to RLG showed no change in 6-NBDG uptake (Fig. 6e), indicating that the persistent changes in mitochondrial oxygen consumption were not simply mediated by increased glucose uptake. Correspondingly, we did not observe any changes in astrocytic glycogen content following RLG (Fig. 6f).

Correlating with our mitochondrial imaging studies, the levels of mitochondrial markers succinate dehydrogenase (SDH) and the voltage-dependent anion channel (VDAC) were not altered by RLG, suggesting no change in total mitochondrial content (ESM Fig. 5). Fatty acid synthase (FAS) and CPT-1a expression were also not altered, indicating that changes in fatty acid-dependency following RLG are likely to be mediated by metabolic flux/activity rather than alternations in expression of enzymes within the FAO pathway.

### Partial recovery of metabolic changes by maintenance of glucose levels

**Fig. 7:**
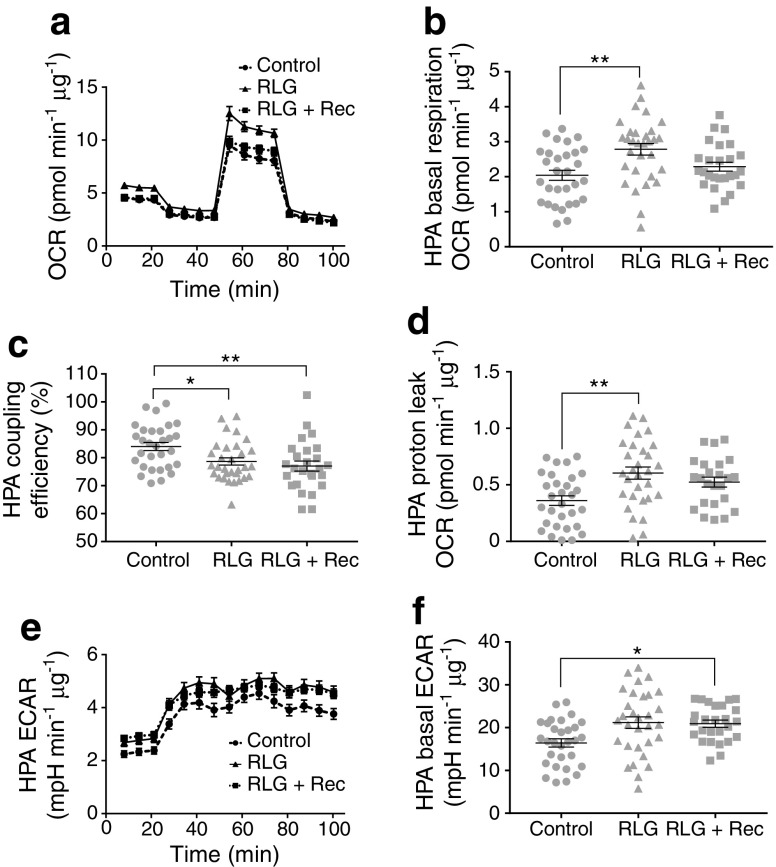
Maintaining glucose levels following RLG partially recovered metabolic adaptations. (**a**) OCR of HPA following RLG with a 4 day recovery after the last low glucose exposure (control, *n* = 31; RLG, *n* = 31; RLG + recovery, *n* = 26). Cells were exposed to oligomycin (10 μmol/l), FCCP (5 μmol/l) and a combination of rotenone and antimycin A (5 μmol/l). (**b**) Mean basal respiration rate (OCR). (**c**) Coupling efficiency calculated from the ratio of oligomycin-sensitive OCR and basal OCR expressed as a percentage. (**d**) Proton leak, calculated from the oligomycin-insensitive (i.e. not ATP synthase-linked) OCR minus non-mitochondrial respiration, from HPA cells; RLG + recovery was not significant vs control or vs RLG. (**e**) ECAR analysis measured during mitochondrial stress tests (control, *n* = 31; RLG, *n* = 31; RLG + recovery, *n* = 26). (**f**) Mean baseline ECAR in HPA cells following RLG and RLG + recovery. Error bars represent standard error of the mean. *p < 0.05, **p < 0.01 vs control. Analyses used unpaired two-tailed Students *t* tests. RLG + Rec, RLG + recovery

To assess whether mitochondrial changes induced by RLG were reversible, we exposed cells to RLG and introduced a 4 day recovery period where glucose levels were maintained at 2.5 mmol/l glucose following the last bout of low glucose exposure. As previously, our RLG treatment group had elevated basal OCR, increased proton leak and elevated basal ECAR (Fig. 7). Following recovery, basal mitochondrial OCR returned to levels comparable with control (Fig. 7a, b). In contrast, coupling efficiency was still reduced (Fig. 7c) and proton leak showed a trend towards recovery, which did not reach statistical significance (Fig. 7d). RLG-induced elevated basal ECAR was largely unchanged following recovery (Fig. 7e, f).

## Discussion

Our studies shed new light on the previously uncharacterised intrinsic metabolic changes in human glial cells in response to acute and recurrent low glucose. We demonstrate that astrocytes, in the absence of physical and chemical signals from neurons, react to low glucose by increasing the activation of the key metabolic sensor AMPK. Changes in AMPK pathway phosphorylation occur within a pathophysiologically relevant glucose concentration range, similar to that which affects CNS glucose-sensing neurons [26].

It is well accepted that astrocytes are more metabolically flexible than neurons, partly because of the higher expression of PFKFB3, which can accelerate glycolysis during metabolic stress [21]. Therefore, to examine specifically the changes in astrocyte metabolism following RLG, we measured markers of mitochondrial and glycolytic metabolism in HPA monocultures. Importantly, we found that daily prior bouts of low glucose (4 × 0.1 mmol/l glucose), lasting 3 h, were sufficient to increase basal mitochondrial and glycolytic metabolism to sustain intracellular ATP supply. Increased basal mitochondrial metabolism is likely mediated, at least in part, by an increased contribution of FAO to basal oxygen consumption. Correlating with this hypothesis, we noted increased mitochondrial proton leak and reduced mitochondrial coupling efficiency in astrocytes exposed to RLG. Importantly, FAO is known to increase uncoupling, as FAO generates increased levels of reactive oxygen species (ROS)/superoxide [27] that can increase proton leak via activation of uncoupling proteins or the ATP/ADP antiporter (ANT; [28]). Previous work has demonstrated that, in vivo, genes for β-oxidation are induced by acute hypoglycaemia, but this fails following recurrent hypoglycaemia [29]. Our data in astrocyte monocultures contrast with these measurements taken from rodent mediobasal hypothalamus, which include both neurons and glia and axon projections tracking through the dissected area. Therefore, it is possible that cell-type-specific changes are masked when measuring mRNA expression from whole-tissue sections.

A previous study demonstrated that low intracellular glucose levels caused by loss of insulin receptors on astrocytes (leading to reduced astrocytic glucose transport) resulted in astrocytic mitochondria that were smaller and less numerous [30], suggesting increased mitophagy. In our study, despite evidence of mitochondrial stress and in contrast to our expectation, examination of the filamentous mitochondrial networks revealed no change in either mitochondrial size or number following acute low glucose or RLG. Indeed, mitochondria exposed to RLG generally displayed a normal filamentous network, with little or no evidence of mitochondrial fragmentation. These data suggest that astrocyte mitochondria successfully adapt to maintain energy production in response to repeated bouts of low glucose availability. It is possible that the relatively acute reductions in glucose availability in our experiments were a mild stress, insufficient to cause gross changes in mitochondrial morphology. Moreover, cells were recovered into solutions containing serum, likely providing sufficient insulin/growth factor receptor activation to aid recovery from the acute stress. Therefore, it is possible that reductions in insulin receptor activation, rather than just intracellular glucose deprivation per se, may mediate the altered mitochondrial morphology reported previously. A similar phenotype of increased mitochondrial fragmentation was also reported using inducible knockdown of insulin-like growth factor 1 (IGF-1) receptors on astrocytes [31], suggesting an important role for hormones in regulating glial cell mitochondrial function that requires further investigation.

Previous studies in rats found no change in brain glycogen levels following recurrent hypoglycaemia [32] or any correlation between brain glycogen content and impaired awareness of hypoglycaemia in humans [33]. However, other studies suggest brain glycogen content is increased following recovery from insulin-induced hypoglycaemia [34] and following recurrent 2-deoxyglucose-induced glucoprivation (reduction in utilisable glucose) in mice [35]. Our data clearly demonstrate that astrocytes intrinsically adapt by increasing cellular metabolism in response to recurrent energy stress. Specifically, rates of extracellular acidification (a marker for glycolysis) following glucose ‘reperfusion’ were elevated in cells exposed to RLG, suggesting an augmented re-activation of glycolysis. However, despite glycolysis being a major determinant of glycogen synthesis, we found no significant change in astrocytic glycogen levels following RLG. Extracellular lactate, which can be generated from glycogen breakdown during aglycaemia [17], increased substantially, relative to glucose availability, during low glucose exposure, though the magnitude of this response was not significantly altered following RLG. We cannot rule out, however, the possibility that more subtle changes in lactate and glycogen occur during the transition to low glucose or on glucose recovery. Moreover, before definitively ruling out a role for astrocytic glycogen and subsequent lactate release, a more replete in vitro model, containing both astrocytes and neurons, should be tested, thus facilitating the inclusion of neurotransmitter dynamics in the model. The ability to produce increasingly credible human neurons and astrocytes from inducible pluripotent stem cell (iPSC) sources could potentially enable such investigations to be performed in an in vitro human system, overcoming some of the physiological limitations of mono-culture systems.

Astrocytes play a key role in maintaining glutamatergic neurotransmission through the glutamate−glutamine cycle (for recent review see Schousboe [36]). Importantly, astrocytic glutamate clearance requires co-transport of three Na^+^ ions [37], meaning that, via the ATP-dependent Na^+^/K^+^ ATPase, there is a significant metabolic cost for astrocytes to maintain their Na^+^ homeostasis, estimated to account for approximately 20% of total astrocytic ATP production [38]. This process may become particularly costly during hypoglycaemia. Moreover, glutamate stimulation of astrocytes produces mitochondrial dysfunction, decreasing mitochondrial spare respiratory capacity and increasing lactate production [39]. Furthermore, recent observations suggest that astrocytic glutamate recycling is diminished following recurrent hypoglycaemia, a change most likely mediated by reduced astrocyte glutamate uptake [40]. Interestingly, recurrent hypoglycaemia is associated with relative GABA excess within the VMH [41]. In addition to glutamate, astrocytes also clear GABA from the extracellular space [42], which is energetically less expensive for the astrocytes than glutamate recycling [43]. This raises the interesting possibility that the glutamatergic failure and relative GABA excess following recurrent hypoglycaemia may be mediated by astrocytes, plausibly as a protective mechanism against excitotoxicity, although further study is required.

Our data add to published findings and suggest that metabolic changes within astrocytes could be protective adaptations to limit brain cell damage or contribute to defective counterregulatory responses following RH. In support of the latter, astrocytes are increasingly recognised as playing an important role in the regulation of whole-body metabolism. For example, chemogenetic activation (by increasing intracellular Ca^2+^) of astrocytes in the arcuate nucleus increases feeding in mice and, conversely, sequestering astrocytic Ca^2+^ decreases food intake [14]. Moreover, astrocytes can be a source of pro-inflammatory cytokines in the brain [44], including TNFα, which has previously been reported to blunt glucose-sensing glucose-inhibited neurons [45], suggesting that pro-inflammatory signals from astrocytes may regulate energy-sensing neurons of the hypothalamus [44]. Accordingly, modulation of inflammatory signalling in astrocytes modulates feeding in response to a high-fat diet [46]. Within the hindbrain, astrocytes can detect hypoglycaemia to regulate adjacent neurons to increase gastric emptying in response to a glucoprivic stimulus [13]. Astrocytes have also been shown to decrease hyperglycaemia in a rodent model of type 1 diabetes [47], suggesting that astrocytes may play fundamental roles in regulating whole-body glucose levels. It should also be noted that there is significant heterogeneity in astrocytes across different brain regions. For example, hypothalamic astrocytes express more genes for lipid oxidation compared with cortical astrocytes, which express more genes for lipid synthesis [48]. Regional differences in astrocytic glycogen content also exist, with high levels in hippocampus and cortex and comparatively lower levels in subcortical structures [49]. Within the hindbrain, only a subset of astrocytes respond to a glucoprivic stimulus [50], indicating that not all astrocytes will respond to a given stimulus in the same manner. Given the significant heterogeneity of neurons within the hypothalamus, it is plausible that subtypes of astrocytes within this region may contribute distinct roles in hypoglycaemia sensing. However, the territory of a single astrocyte is likely to overlap with multiple neuronal types, probably requiring subcellular site-specific responses.

In conclusion, our data further support the evidence that glial cells detect hypoglycaemic stress. Moreover, we demonstrate that mitochondrial function (increased FAO) in human astrocytes is altered by RLG. These data suggest a possible role for glial mitochondrial adaptations in defective glucose counterregulation or protection against hypoglycaemia, and highlight the need for more investigation into neuron-glial interactions during and following recurrent hypoglycaemia.

## Electronic supplementary material

ESM (PDF 14158 kb)

## Data Availability

The datasets generated during and analysed during the current study are available from the corresponding author on reasonable request.

## Abbreviations

ACC: Acetyl-CoA carboxylase
AMPK: AMP-activated protein kinase
CPT1: Carnitine palmitoyltransferase 1
CRR: Counterregulatory response
ECAR: Extracellular acidification rate
FAO: Fatty acid oxidation
FCCP: Carbonyl cyanide 4-(trifluoromethoxy)phenylhydrazone
GFAP: Glial fibrillary acidic protein
HK: Hexokinase
HPA: Human primary astrocyte
MPC: Mitochondrial pyruvate carrier
6-NBDG: 6-(*N*-(7-nitrobenz-2-oxa-1,3-diazol-4-yl)amino)-6-deoxyglucose
OCR: Oxygen consumption rate
PFK[P]: Phosphofructokinase [platelet]
RLG: Recurrent low glucose
VDAC: Voltage-dependent anion channel
VMH: Ventromedial nucleus of the hypothalamus
