# Supplementary material for: Basal fatty acid oxidation increases after recurrent low glucose in human primary astrocytes
Source: Diabetologia. 2018 Oct 6;62(1):187–98. doi: 10.1007/s00125-018-4744-6 (PMC6290858; doi:10.1007/s00125-018-4744-6)
Supplement: Supplementary file 1 — (PDF 14158 kb) [file 125_2018_4744_MOESM1_ESM.pdf]

ESM methods

Cell culture

Human primary astrocytes were maintaining in stock media (DMEM; #31885, Thermo Fisher Scientific, Loughborough, UK) containing: 5.5 mmol/l glucose; 10% (vol/vol) FBS (no. 10270-106 Thermo Fisher Scientific) and gentamicin (25 µg/ml; no. 15750-060, Thermo Fisher Scientific). To enhance adherence of cells during growth, flasks and petri dishes containing HPA cells were pre-coated with poly-L-lysine (20 µg/ml; Sigma, no. P1274-25MG) in dH_2O. 1 x 10^6 cells were seeded into T75cm^2 flasks and allowed to grow until passaging after approximately 7-10 days. For experiments, HPA cells were seeded into 60 mm round petri dishes in HPA stock media the day prior to study; whereas U373 cells were seeded into DMEM 11966 media supplemented with 7.5 mmol/l glucose and FBS. U373 cells, were maintained in U373 stock media (DMEM; no. D5671, Sigma-Aldrich, Gillingham, United Kingdom) containing 25 mmol/l glucose, supplemented: FBS (no. 10270-106, Thermo Fisher Scientific); L-glutamine (4 mmol/l); penicillin/streptomycin (100 U/ml; 100 µg/ml, Thermo Fisher Scientific).

Immunoblotting

Primary antibodies used were (from Cell Signaling Technologies): pThr172 AMPK (1:1,000; no2535, rabbit), pSer79 acetyl-CoA carboxylase (ACC; 1:1,000; no. 3661, rabbit), hexokinase I (HKI; 1:1,000; no 2024), hexokinase II (HKII; 1:1,000; no 2867, rabbit), phosphofructokinase-platelet-type (PFK(P); 1:1,000; no.5412, rabbit), succinate dehydrogenase A (SDHA; 1:1,000; no 11998, rabbit). Mouse β-actin (1:10,000; no. NB600-501) was from Biotechne, Abingdon, UK. Carnitine palmitoyltransferase 1-a (CPT1a; 1:1,000; no. 15184-1-AP, rabbit) was from Proteintech. Fatty acid synthase (FAS; 1:1,000; no. 3180S, rabbit) was from New England Biotech. Hexokinase III (HKIII; 1:1,000; no. ab126217, rabbit)

was from Abcam. Glucose transporter 1 (Glut-1; 1:1,000; no. 07-1401, rabbit) was from Millipore. Proteins were visualised and quantified using the Odyssey scanner (Licor, Cambridge, UK). Secondary antibodies used included Alexa fluor™ 680 goat anti-mouse IgG (A21057, Thermo Fisher Scientific), and goat anti-rabbit IgG IRDye800® (ROCK611-132-122, VWR).

Analysis of cellular metabolism

Assay plates were loaded into the extracellular flux analyser and studied on a 3-minute mix, 3-minute measure cycle and compounds injected as indicated. Oligomycin (10 μM) was added to block ATP synthase, FCCP (5 μM) to stimulate maximal respiration, and rotenone and antimycin A (R/A; 5 µM each) were added to inhibit complex I and III, respectively. Following assay completion, the assay plate was removed from the analyser, media removed and replaced with 100 µl of 50 mmol/l NaOH to lyse cells. Protein concentrations were determined by Bradford assay using NaOH as a vehicle. Cellular oxygen consumption rate (OCR) and extracellular acidification rate (ECAR) were normalised to total protein concentration of each well and where appropriate, normalised to baseline values (prior to compound injection). Samples with negative values for oxygen consumption rate (likely due to the presence of an air bubble in the microchamber) were excluded from analysis.

Fluorescent imaging

HPA cells were plated on poly-L-lysine coated round glass coverslips (12 mm) in 24 well plates at 50,000 cells per well and exposed to 4 days of recurrent hypoglycemic conditions as earlier described. Cells were loaded for 15 minutes with MitoTracker Red CMXRos (50 nM; Molecular Probes, M7512, no. 1785958) in 2.5 mM glucose DMEM prior to the final low glucose exposure, and fixed with 4% paraformaldehyde and 0.25% glutaraldehyde for 20

minutes after treatment. Cells were permeabilised with lysine buffer (PBS, 10% normal donkey serum, 50 mM L-lysine, 0.2% Triton X-100) and stained with DAPI nuclear stain (Molecular Probes, no. D1306, 0.2mg/mL in PBS). Coverslips were mounted in fluoroshield mounting medium (Abcam, no. ab104135, #GR311329-1). Z-stacks of cells were taken using confocal microscopy (Leica DMi8; x63/oil immersion lens). Maximum projections were created using LAS X software (Version 1.9.0.13747). Images were thresholded, and the mean, median and total number of objects per image calculated using a custom written MatLab script. Data from the script were analyzed using one-way ANOVA with post-hoc Bonferroni in GraphPad Prism (Version 5.01). For vimentin staining, cells were permeabilised with lysine buffer and incubated overnight with an anti-vimentin antibody (Sigma, V6630, no. 102M4831, mouse, 1:500 in lysine buffer). Cells were incubated in anti-mouse secondary for 1 hour (Alexa fluor 568, Thermo Fisher Scientific, A10037, no.1827879 1:500 in PBS) and co-stained with DAPI. Coverslips were mounted in fluoroshield mounting medium and cells imaged by confocal microscopy (Leica DM4000 B LED; x40). For GFAP staining, cells were cultured for 3 hours in either 2.5 or 0.1 mM glucose supplemented with 1% (v/v) FBS and 22.5 or 24.9 mM mannitol. Cells were then fixed with 4% paraformaldehyde and 0.25% glutaraldehyde for 20 minutes after treatment. Cells were permeabilised lysine buffer and incubated overnight with an anti-GFAP antibody (Dako, z0334, no. 20035994, rabbit, 1:100 in lysine buffer). Cells were incubated in anti-rabbit secondary for 1 hour (Alexa fluor 488, Thermo Fisher Scientific, A21206, no. 1927937 1:500 in PBS) and co-stained with DAPI. Coverslips were mounted in fluoroshield mounting medium and cells imaged by fluorescence microscopy (Leica DM4000 B LED; x40).

ESM Fig. 1

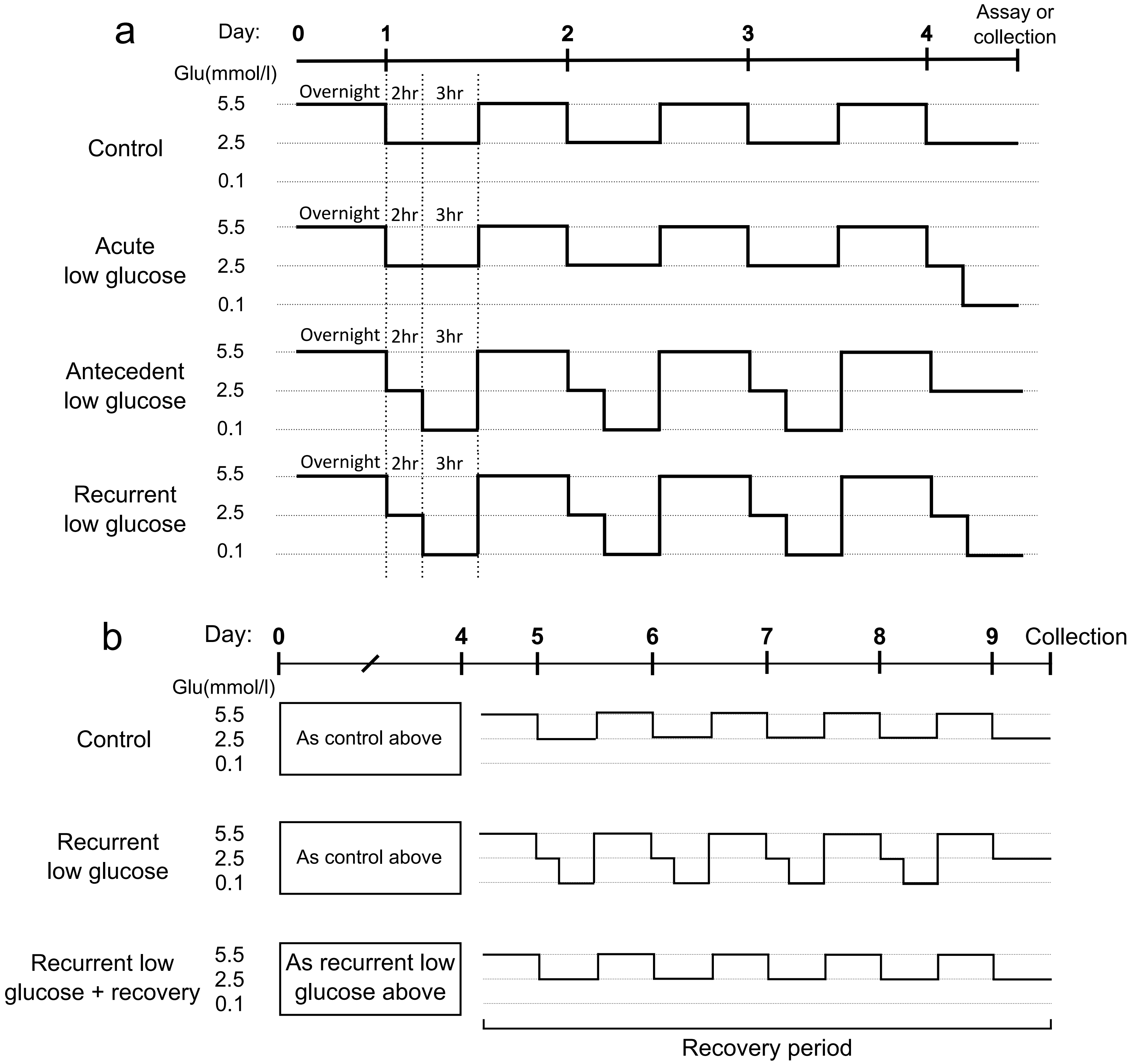

ESM Figure 1. Illustration of the recurrent low glucose model. Over 4 days, cells are exposed to 0, 1, 3 or 4 bouts of 0.1 mmol/l glucose, representing control, acute low glucose, antecedent low glucose or recurrent low glucose, respectively.

ESM Fig. 2

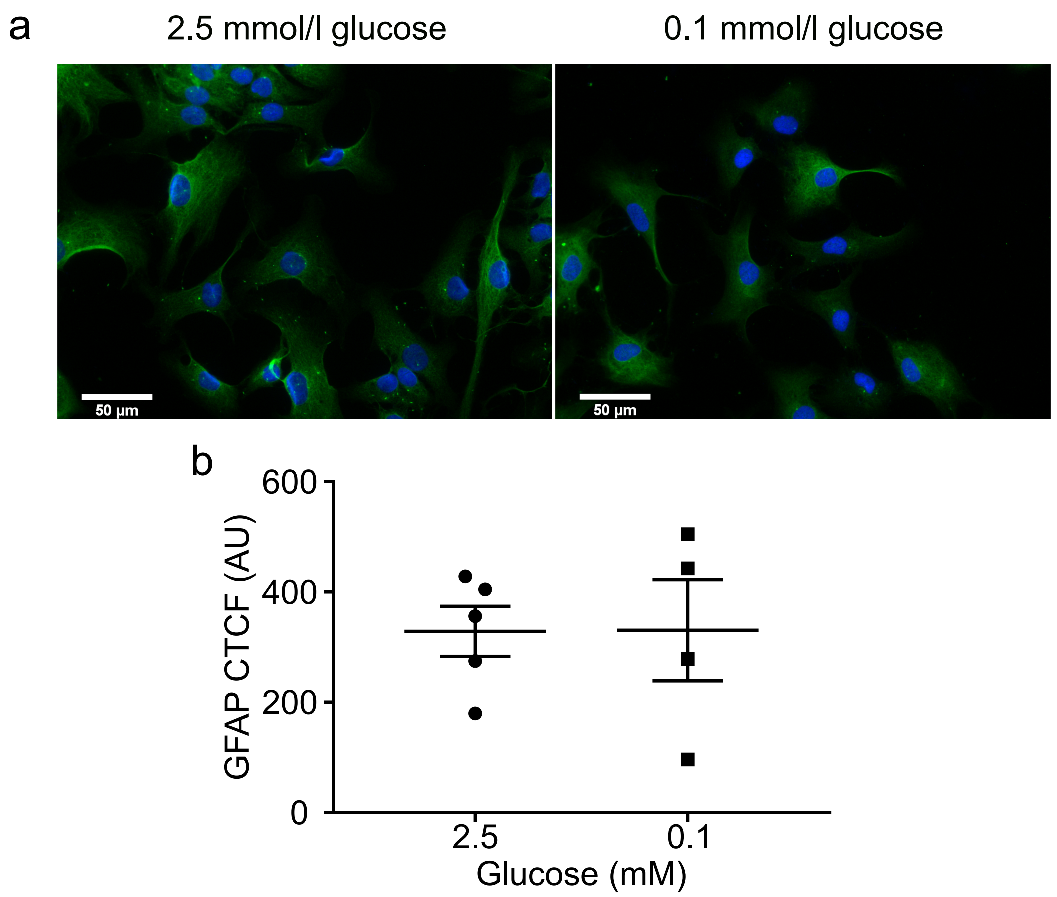

ESM Figure 2. Human primary astrocytes (HPA) are GFAP positive.
A. HPA cells staining with a GFAP antibody (green) and DAPI to identify nuclei (blue). Scale bar: 50 μm. B. Quantified correct total cell fluorescence (CTCF) of GFAP immunofluorescence (control, n=5; low glucose, n=4). Unpaired two-tailed student’s t- test. Error bars represent standard error of the mean.

ESM Fig. 3

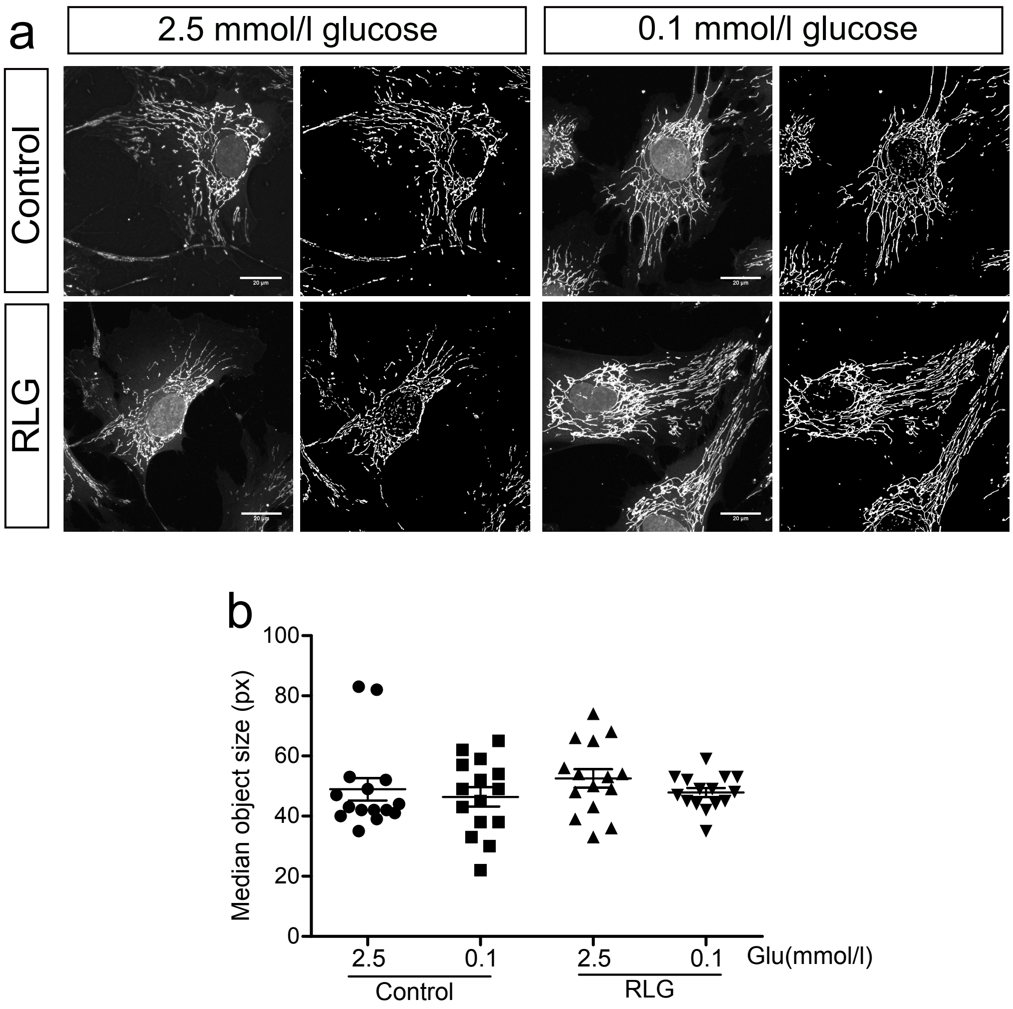

ESM Fig 3. Acute and recurrent low glucose did not alter mitochondrial number or length. A. Representative confocal images of human primary astrocytes (HPA) cells exposed to 2.5 or 0.1 mmol/l glucose for 3 hours following control or recurrent low glucose exposure (RLG). Raw confocal images are shown on the lefthand side, with extracted signal images shown on the right. Quantification of median object size (in pixels) using a custom MatLab script (15 images across 3 separate experiments; scale bar 20 µm). One-way ANOVA with post hoc Bonferroni multiple comparisons tests. Error bars represent the standard error of the mean.

ESM Fig. 4

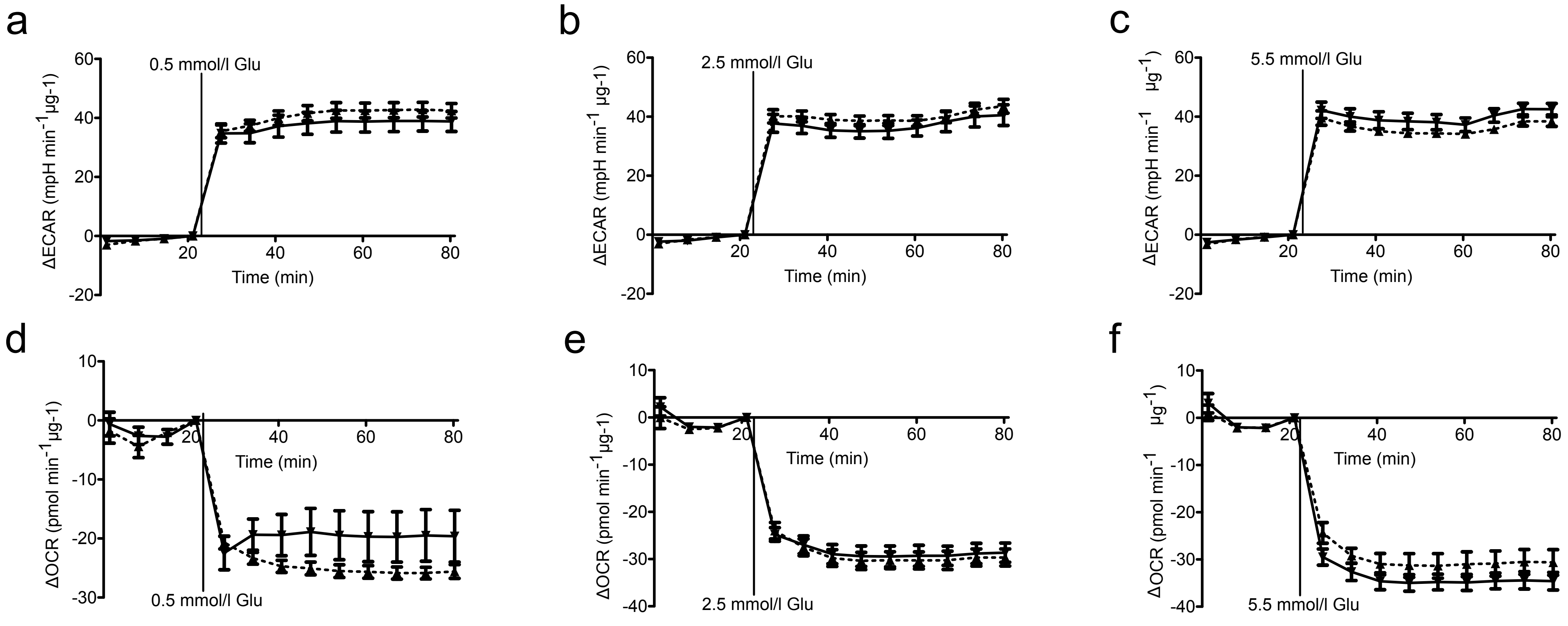

ESM Figure 4. Recurrent low glucose (RLG) did not alter glycolytic re-activation after acute low glucose exposure in U373 cells. Extracellular acidification rates (ECAR) increased in U373 cells on re-introduction of 0.5 mmol/l glucose (A), 2.5 mmol/l glucose (B) and 5.5 mmol/l glucose (C) although this was not significantly different between control and recurrent low glucose (RLG) treated cells. Oxygen consumption rate (OCR) decreased on addition of 0.5 mmol/l glucose (D), 2.5 mmol/l glucose (E) and 5.5 mmol/l glucose (F), which was not significantly different between control and RLG (control, n=10-12; RLG, n=9-11). Two-way ANOVA with post hoc Bonferroni multiple comparisons tests. Error bars represent standard error of the mean.

ESM Fig. 5

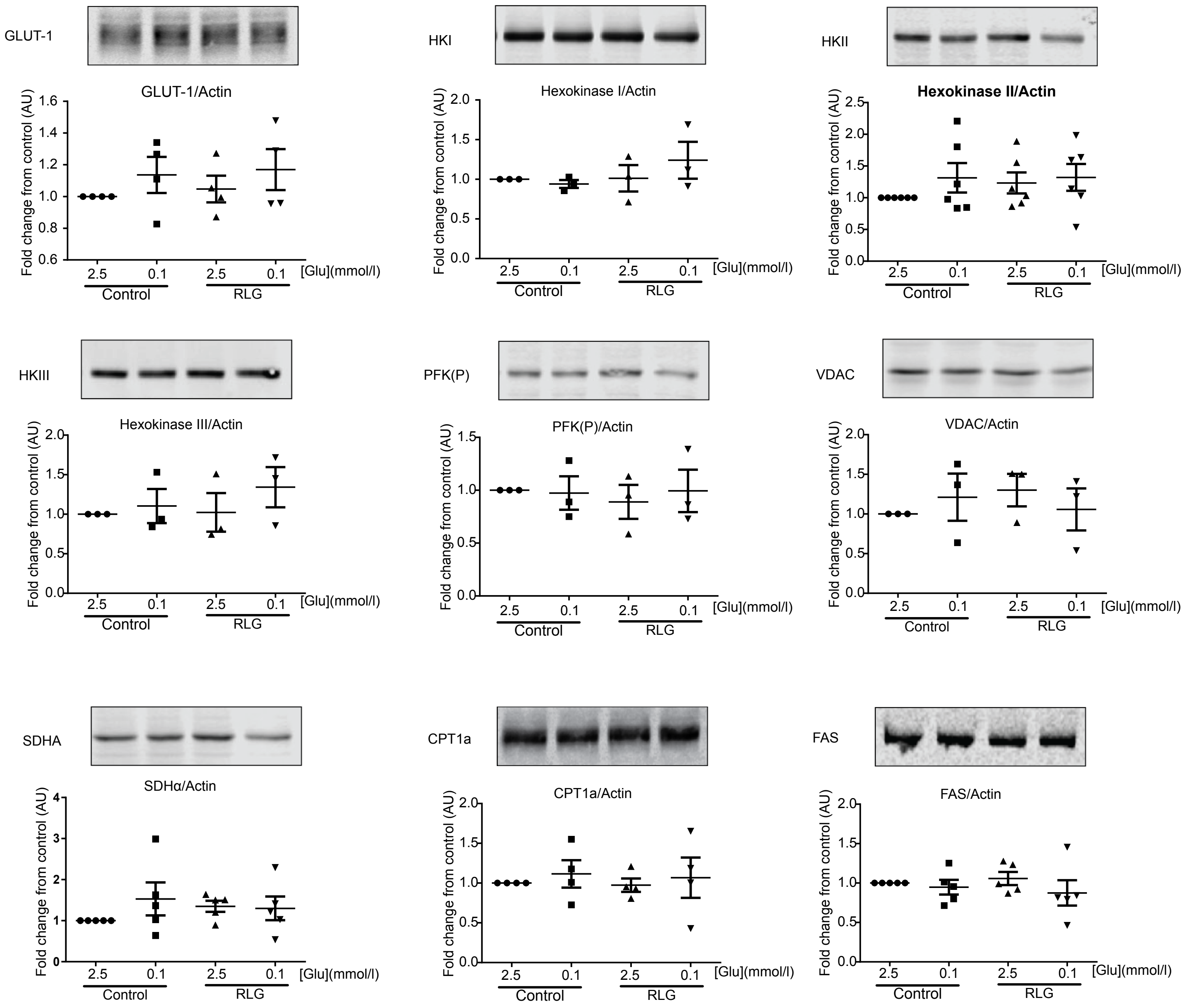

ESM Figure 5. Expression of key mitochondrial and glycolytic markers were not altered by acute or recurrent low glucose exposure (RLG). Glucose transporter 1 (GLUT-1), hexokinase I (HKI), hexokinase II (HKII), hexokinase III (HKIII), phosphofructokinase [platelet; PFK(P)], voltage dependent anion channel (VDAC), succinate dehydrogenase A (SDHA), carnitine palmitoyltransferase 1a (CPT1a) and fatty acid synthesis (FAS) were not altered by acute or recurrent low glucose exposure (n=3 -4). One-sample t-tests in comparison to control. Error bars represent standard error of the mean.
